# The Root-Associated Microbial Community of the World’s Highest Growing Vascular Plants

**DOI:** 10.1007/s00248-016-0779-8

**Published:** 2016-05-31

**Authors:** Roey Angel, Ralf Conrad, Miroslav Dvorsky, Martin Kopecky, Milan Kotilínek, Inga Hiiesalu, Fritz Schweingruber, Jiří Doležal

**Affiliations:** Max Planck Institute for Terrestrial Microbiology, Karl-von-Frisch-Str. 10, Marburg, Germany; Division of Microbial Ecology, Department of Microbiology and Ecosystem Science, University of Vienna, Althanstrasse 14, Vienna, Austria; Institute of Botany, The Czech Academy of Sciences, Zámek 1, 25243 Průhonice, Czech Republic; Department of Botany, Faculty of Science, University of South Bohemia, Na Zlate stoce 1, 37005 Ceske Budejovice, Czech Republic; Swiss Federal Research Institute WSL, Zuercherstrasse 111, 8903 Birmensdorf, Switzerland

**Keywords:** Vascular plants, Upward migration, Subnival soil, Plant-associated bacteria

## Abstract

Upward migration of plants to barren subnival areas is occurring worldwide due to raising ambient temperatures and glacial recession. In summer 2012, the presence of six vascular plants, growing in a single patch, was recorded at an unprecedented elevation of 6150 m.a.s.l. close to the summit of Mount Shukule II in the Western Himalayas (Ladakh, India). Whilst showing multiple signs of stress, all plants have managed to establish stable growth and persist for several years. To learn about the role of microbes in the process of plant upward migration, we analysed the root-associated microbial community of the plants (three individuals from each) using microscopy and tagged amplicon sequencing. No mycorrhizae were found on the roots, implying they are of little importance to the establishment and early growth of the plants. However, all roots were associated with a complex bacterial community, with richness and diversity estimates similar or even higher than the surrounding bare soil. Both soil and root-associated communities were dominated by members of the orders Sphingomonadales and Sphingobacteriales, which are typical for hot desert soils, but were different from communities of temperate subnival soils and typical rhizosphere communities. Despite taxonomic similarity on the order level, the plants harboured a unique set of highly dominant operational taxonomic units which were not found in the bare soil. These bacteria have been likely transported with the dispersing seeds and became part of the root-associated community following germination. The results indicate that developing soils act not only as a source of inoculation to plant roots but also possibly as a sink for plant-associated bacteria.

**Electronic supplementary material:**

The online version of this article (doi:10.1007/s00248-016-0779-8) contains supplementary material, which is available to authorized users.

## Introduction

One of the most apparent consequences of global warming is the upward migration of plants to higher elevations, from which they were previously excluded [1]. In some cases, plants have been able to migrate to entirely unvegetated areas in the subnival zone, which were previously too cold to permit growth or only recently exposed due to glacial recession (e.g. [2–4]). These subnival soils are usually very young and poor. Soil development from the eroded bedrock is largely a combination of physicochemical processes, such as cryoturbation and mineral leaching, and of microbial processes (including chemical weathering and nitrogen fixation) [5].

Since microorganisms are easily dispersed by wind and, on the whole, more stress-tolerant than plants, they colonise subnival zones before plants do. Several studies have documented the presence of bacteria [6–8], archaea [7, 9, 10] and fungi [7] in subnival zones and glacial forefields within a few years from deglaciation and have monitored the subsequent succession of microbial communities. Whilst the particular bacterial community structure varied by site, some common early colonisers in the forefields of receding glaciers from different regions worldwide included members of the phyla alpha-, beta-, gamma- and deltaproteobacteria, Bacteroidetes, Cyanobacteria, Actinobacteria and Acidobacteria. Although these phyla are typical also of mature soils in temperate regions, their proportions in subnival zones differed significantly from mature soils and were composed of high proportions of Betaproteobacteria and Cyanobacteria, for example. In the absence of plants, biological soil crusts develop in subnival zones and photosynthesis and nitrogen fixation are mainly carried out by Cyanobacteria and other microorganisms living in the crust [10–13].

With the subsequent colonisation and establishment, vascular plants further promote the processes of soil development by enriching the soil with organic matter through rhizodeposition and foliage and by promoting weathering of the bedrock [14]. Much like microbes, specific plants are known to be particularly suitable as pioneers of subnival zone soils [15, 16]. These often include compact and wind- and frost-tolerant species, which are able to grow on poor soils and during short growing seasons [17]. The portion of the soil directly surrounding the plant roots, known as the rhizosphere, and the root surface itself (rhizoplane) are a hot spot for microbial activity, which is stimulated by the added carbon provided by root exudates and sloughing of cells [18]. This plant-mediated stimulation of microbial activity in the rhizosphere speeds up soil formation. However, the rhizosphere and rhizoplane are not simply sites of stimulated activity; rather, plants also select for a specific root-associated microbial community, which differs from the surrounding bulk soil [19]. The root-associated bacterial community composition may differ with plant species [20], though it is commonly dominated by alphaproteobacteria (such as *Rhizobia*), betaproteobacteria (e.g. *Burkholderia*), gammaproteobacteria (such as *Pseudomonas*), Firmicutes (e.g. *Bacillus*) and Bacteroidetes [21].

**Fig. 1:**
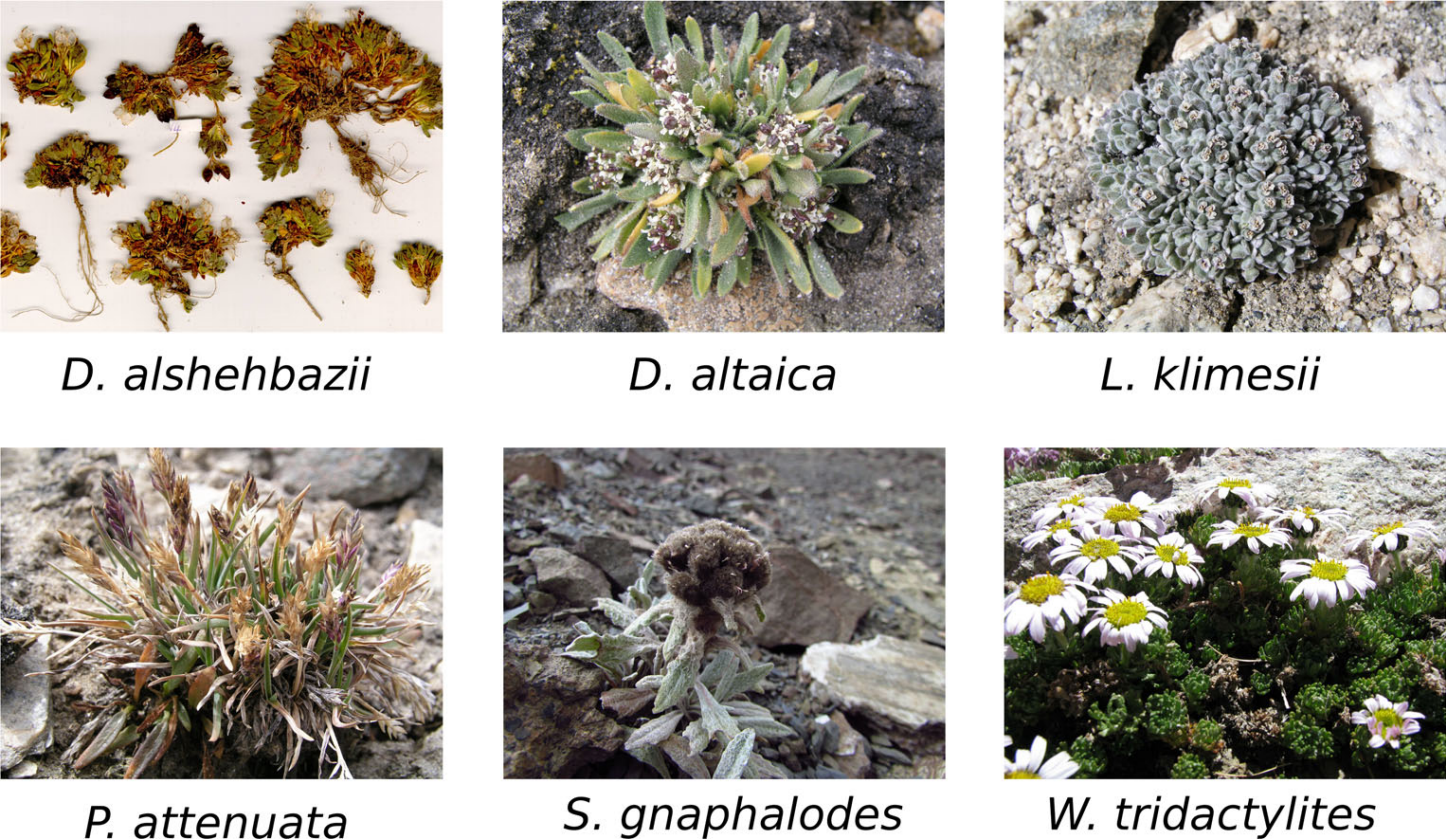
Six vascular plant species discovered at 6150 m.a.s.l.: *Draba alshehbazii*, *Draba altaica*, *Ladakiella klimesii*, *Poa attenuata*, *Saussurea gnaphalodes* and *Waldheimia tridactylites*

The Northwestern Himalayas is an extensive and thinly populated high-altitude cold desert characterised by low temperatures and low precipitation owing to its location in the Himalayan rain shadow [22]. This region has been experiencing an accelerated rise in temperature with rapidly retreating glaciers during the 1990s [23, 24], whilst over the last decade there has been a general shift towards rainy (often snowy) summers, a prolonged growing season and unpredictable extreme precipitation events [25]. In an expedition performed in the summer of 2012 to study the plant diversity and the soil microbiology of the region, we have recorded a small and sparse patch of vegetation close to the summit of Mount Shukule II, India, at an unprecedented elevation of 6150 m.a.s.l. In this patch, we identified six vascular species: *Draba alshehbazii*, *Draba altaica*, *Ladakiella klimesii*, *Poa attenuata*, *Saussurea gnaphalodes* and *Waldheimia tridactylites* (Fig. 1).

In this study, we collected three individual plants from each species. Using barcoded Illumina MiSeq amplicon sequencing of the SSU rRNA gene, we characterised the root-associated bacterial community of the plants and that of the adjacent bare soil. In addition, we analysed the chemical composition of the bare soil and plant tissues and determined the plant age. We hypothesised that since microorganisms precede plants in colonising subnival soils and are required by plants for the supply of minerals and fixed nitrogen, the root-associated bacteria of the plants will be a selected subset of the community found in the soil, particularly comprising organisms capable of nitrogen fixation.

## Materials and Methods

### Study Site and Sampling Technique

The presence of six vascular plant species was recorded at an unprecedented elevation of 6150 m.a.s.l. close to the summit of Mount Shukule II in the Northwestern Himalayas (southwest spur of the Tibetan Plateau in Eastern Ladakh, Jammu and Kashmir State, India), 15 km east of Tso Moriri Lake (32°59′N, 78°30′E; Fig. S1). The plants were growing in a single and small patch on a southwest-facing small boulder field (approx. 1 ha) on an undeveloped calcareous soil. The region is arid (Leh: 115 mm year^−1^, 3514 m.a.s.l., approx. 170 km northwest of the study region; Gar: 54 mm year^−1^, 4232 m.a.s.l., approx. 160 km southeast of the study region) because most of the summer monsoon precipitation is blocked by the main Himalaya Range, as well as the range of Hindu Kush stands as a barrier to the westerly disturbances, which bring winter precipitation to this region. A TMS data logger (see below) was installed on the spot in 2011, and on 15 August 2012 three individuals from each species were sampled with intact root systems as possible. The roots were separated from rhizospheric soil (by shaking and washing in glacial water), cut from the shoot and dried in clean paper bags. In addition, dry bare soil (0- to 5-cm depth) from the same site (approx. 1 m apart from the patch) was also sampled in six replicates for comparison and stored in clean paper bags until further analysis.

### Plant Species

The following species were studied in this work (www.efloras.org/) [26]:

*L. klimesii* (Al-Shehbaz) D. A. German & Al-Shehbaz (Brassicaceae) is a perennial herb forming compact cushions 1–3 cm tall and 2–10 cm in diameter. It has a single unbranched tap root with few lateral fine roots. The species is endemic to the western part of the Tibetan Plateau (E. Ladakh, W. Xizang) and grows in subnival zones near the snowline. In Ladakh, this species occurs from 5350 to 6150 m.a.s.l., with optimum around 5800 m.a.s.l.

*D. altaica* (C. A. Mey.) Bunge (Brassicaceae) is a perennial herb forming compact cushions 2–8 cm tall and 1–5 cm in diameter, with a main tap root and many fine lateral roots. Its distribution range encompasses the continental Central Asiatic mountains from southern Siberia and Kazakhstan to Mongolia. It grows in rock crevices, on stabilised slopes, on moraines and at streamsides. In Ladakh, the species occurs at 3420–6150 m.a.s.l., with optimum around 5500 m.a.s.l.

*D. alshehbazii* Klimeš & D. German (Brassicaceae) is a perennial herb forming compact cushions 1–2 cm tall and 1–5 cm in diameter, with a main tap root and many fine lateral roots. So far, the species is known from several localities in eastern Ladakh, but its presence in China (W. Xizang) is also expected. It is restricted to subnival zones at altitudes from 5470 to 6000 m.a.s.l., with most localities situated above 5700 m.a.s.l.

*W. tridactylites* Kar. & Kir. (Asteraceae) is a perennial herb, 1–5 cm tall, with a thick woody procumbent rhizome bearing many prostrate branches with few adventitious roots. Its distribution range encompasses continental Central Asiatic mountains from Kazakhstan to Mongolia. It prefers wet gravel screes, mesic slopes and rock crevices. In Ladakh, this species occurs from 3600 to 6150 m.a.s.l., with optimum between 4500 and 5000 m.a.s.l..

*S. gnaphalodes* (Royle) Sch. Bip. (Asteraceae) is a perennial herb, 2–6 cm tall, forming low-density stands of rosettes, borne on long unrooted belowground stems which grow from a main root. Its distribution range encompasses continental Central Asiatic mountains from E. Kazakhstan to Tibet. It grows in screes and on unstable solifluction soils. This species grows in Ladakh at 4650–6150 m.a.s.l.., with optimum between 5200 and 5600 m.a.s.l.

*P. attenuata* Trin. (Poaceae) is a perennial grass, 3–30 cm tall, forming dense tussocks with adventitious root system. Its distribution range reaches from southern Siberia and continental Central Asia to humid East Himalayas. It grows on stony steppes, in dry river beds, in screes, on mesic turf and alpine slopes. The species grows in Ladakh from 3250 to 6150 m.a.s.l., with optimum between 5000 and 5500 m.a.s.l.

All six species are known to produce large amounts of wind-borne seeds.

### Climate Measurement

We used TMS loggers (TOMST) to record soil (10-cm depth), surface and air (10 cm above) temperature, and average soil moisture down to 10-cm depth (utilising Time Domain Transmission principle). Volumetric water content was calculated from raw Time Domain Transmission measurements according to a formula developed for coarse-grained soils [27]. The loggers were recording hourly values from August 2011 to September 2012. Furthermore, we measured air temperature (*T*) and relative humidity (RH) using a HOBO U23 Pro v2 logger (Oneset) at 5900 m.a.s.l. from August 2008 to June 2013 (3 cm aboveground, shaded). RH values with a daily mean above 80 % were used as a proxy for snowfall [17].

### Chemical Characterisation of the Soil and Plant Samples

Major cations (Ca^+2^, Mg^+2^, K^+^ and Na^+^) as well as nitrogen and phosphorous species were measured in all soil samples (rhizospheric and bare). Cations were quantified through atomic absorption spectroscopy using SpectrAA 640 (Varian Techtron) at the Analytical Laboratory of the Institute of Botany, Czech Republic. Ammonia, nitrate and total nitrogen were determined colorimetrically after Kjeldahl mineralization using automatic FIAstar 5010 Analyzer (Tecator). Phosphorous was determined colorimetrically after digestion in HClO_4_ using SHIMADZU UV-1650PC spectrophotometer. To assess the plants’ physiological condition, leaf *δ*^13^C value, and fructan and free sugar (simple sugars) contents were measured. *δ*^13^C values in the plant leaves, as well as total carbon and nitrogen in the plant leaves and soil, were measured using an elemental analyser coupled to an IRMS at the Stable Isotope Facility, UC Davis, USA (stableisotopefacility.ucdavis.edu). *δ*^13^C values were used as an integrated, long-term measure of the ratio between internal and ambient CO_2_ concentrations (Ci/Ca), which reflects the intrinsic water use efficiency of the plants [28]. Fructan content was determined colorimetrically, and free sugars were quantified using a high-performance anion exchange chromatography with a pulsed amperometric detection [29]. For that we used a Dionex ICS-3000 system with electrochemical detector and a Dionex CarboPac PA1 column.

### Anatomical Age Determination

Plant ages were determined by ring counting in the oldest sections in the transition between the hypocotyl and the primary root (root collar). In this zone, all annual rings of perennial plants exist and the reaction to mechanical stress caused by growth seems to be reduced to a minimum [30]. Prior to sectioning, samples were stored in 40 % ethanol. Then, transverse, tangential and radial sections were cut from all studied individuals using a sliding microtome, stained with Astra Blue and Safranin and embedded in Canada Balsam. Microscopic images of these sections were made for counting of the consecutive number of rings and anatomical descriptions [31, 32].

### Arbuscular Mycorrhizal Fungi Identification

The presence of arbuscular mycorrhizal fungi (AMF) and dark septate fungi (dark septate endophyte, DSE) in the roots of the studied plants was determined using grid-line intersection light microscopy [33].

### DNA Extraction and Illumina MiSeq Sequencing

DNA was extracted from the roots of each of three replicate plants and from six replicate bare soil samples. For the root samples, the entire dry root system, clean from soil particles (weighing <0.1 g in total), was packed into a Lysing Martix E tube (MP Biomedicals), whilst for the soil samples 0.4 g (dry weight) was used. Extractions were then performed according to a previously published protocol [34, 35] and the pellets were resuspended in either 50 (root samples) or 200 ml (soil samples) of low TE buffer (10 mm Tris–HCl and 0.1 mm EDTA). DNA extracts were checked for quality by electrophoresis and were quantified using Qubit dsDNA HS Assay Kit (Life Technologies). The extracts were used directly as DNA templates for PCR reactions. The root-associated bacterial communities were identified through sequencing of rRNA gene fragments using barcoded high-throughput sequencing [36]. Altogether, we sequenced 24 PCR products (18 from the plant roots + 6 bare soil samples). We used primers 343Fmod (5′-TACGGGWGGCWGCA-3′) and 784Rmod (5′-GGGTMTCTAATCCBKTT-3′) targeting 446 bp in the V3 and V4 regions of the 16S rRNA gene [37]. A set of 24 forward primers each containing a unique 8-bp barcode and one reverse primer were synthesised to serve as sample identifiers (Table S1). PCR amplifications were done in triplicate using the following mixture: each PCR reaction was 25 μl in volume and contained 2.5 μl 10× AccuPrime™ PCR buffer I, 1.5 mM MgCl_2_, 0.5 μl of Taq DNA polymerase (Invitrogen), 0.25 μM of each primer (Sigma-Aldrich) and 1 μl of DNA template. The following programme was used for amplification: 94 °C for 5 min followed by 28 cycles of 94 °C for 30 s, 52 °C for 30 s, and 68 °C for 30 s and a single step of final elongation at 68 °C for 10 min. Following amplification, the samples were pooled and purified using QIAquick PCR Purification Kit (Qiagen). Library preparation and sequencing services were provided by GATC Biotech (Germany). Paired-end sequencing was performed on a MiSeq platform (Illumina) using the v2 Reagent Kits (Illumina). The raw fastq files were deposited into NCBI’s SRA database (http://www.ncbi.nlm.nih.gov/sra) and can be found under BioProject accession number PRJNA285086.

### Sequence Data Processing and Statistical Analysis

Processing of raw sequence data was done using mothur v.1.32.1 [38]. In general, we followed the standard operating procedure for MiSeq datasets described by Kozich and colleagues [36] for assembling contigs from the two fastaq files, removing low-quality sequences, trimming the sequences to remove primer and barcode parts, removing singleton reads, and for filtering and removing non-overlapping alignment regions. Sequence alignment and classification were done using SINA V1.29 [39] against the SILVA 115 SSU NR99 database [40]. De novo chimera detection was done using UCHIME [41] and pre-clustering of sequences with up to three bases difference was done using a pseudo-single linkage algorithm according to Huse and colleagues [42]. Operational taxonomic units (OTUs) were assigned based on an uncorrected pairwise matrix which was clustered based on a 97 % sequence similarity level using an average neighbour linkage algorithm [43]. Community richness was estimated using the non-parametric Ace estimator and the best-fitted parametric estimator using CatchAll [44], whilst diversity was estimated using Shannon–Wiener, inverse Simpson and Berger–Parker indices (all using mothur). Error propagation for the richness estimators was done using the R package ‘propagate’ [45, 46]. To account for sample size differences, all samples were subsampled (rarefied) to the minimum sample size (10,122 sequences) using bootstrap subsampling at 1000 iterations, for all downstream analyses. Sample means (of richness and diversity estimates) were compared using ANOVA (assuming equal variance) and the differences were tested using Tukey’s HSD. Soil and root-associated communities were clustered into metacommunities using Dirichlet multinomial mixtures modelling [47]. For that purpose, the R package ‘DirichletMultinomial’ [48] was used to consecutively fit Dirichlet mixture models with one to seven components and to derive the corresponding relative abundances of each OTU in the postulated metacommunities. The model fit was calculated using the Laplace approximation to the negative log model evidence, plotted against the number of tested models (*K*), and the minimum was chosen as the most appropriate model for the data.

## Results

### Summary of Climate Conditions at 6150 m

The mean annual *T* of the soil in the studied period was −8.7 °C and absolute minimum air *T* was −36 °C (10 January 2012), whilst absolute maximum air *T* was 28.2 °C, measured on 15 June 2012. On that day, the maximum daily range of 42.4 °C was also recorded (min, −14.1 °C; max, 28.3 °C). The vegetation season—defined either as the period with mean daily air, surface or soil temperatures above zero—lasted 82, 88 and 89 days, respectively, with mean temperature values of 2.71, 3.62 and 2.97 °C (mean soil moisture 16.2 % during 89 days) between mid-May and mid-September. Although this may seem to be a long vegetation season, the numbers of days with mean soil or surface temperature above 5 °C were only 12 and 17 (mean of 5.93 and 6.56 °C), respectively. We assume that plant growth was limited almost exclusively to this short period. Freezing of the soil is more critical for vascular plants than aboveground frost. Whilst air temperature during the growing season usually dropped below zero at night for more than 10 h (regularly to −7 °C in the morning hours, absolute minimum of −16.2 °C at 4:30 a.m. on 17 June 2012), soil temperatures never dropped much below zero and remained between +3 and −1 °C (Fig. S2). Above 5800 m.a.s.l., practically all precipitation falls as snow and it can occur all year round. The snowfall during the summer is typically very little in each event (0–3 cm) and melts after a few hours. Based on relative air humidity as a proxy for snowfall, the total number of days with either a snowfall or with a continuous snow cover was 273 in 2009, 228 in 2010, 132 in 2011 and 141 in 2012.

### Chemical Characterisation of the Samples

All rhizospheric soils (i.e. soil shaken off from the root systems) had similar concentrations of major cations and phosphate, which were in turn similar to the bare soil (Table 1). However, some differences in nitrogen species were notable. The bare soil samples had a much higher concentration of ammonium compared to the rhizospheric soils (6.2 vs. 1.1 mg kg^−1^). The rhizospheric soil of *P. attenuata* had exceptionally high nitrate and total nitrogen values (10.7 and 651 mg kg^−1^, respectively). In contrast, no notable differences in the carbon or nitrogen content of the plant tissues were observed. Stable carbon isotope ratios were also similar for all plants, with *δ* values ranging between −25.2 and −27.4. Free sugar and fructan concentrations were relatively low, except in *W. tridactylites* and *S. gnaphalodes* (free sugars and fructan, respectively).

**Table 1:** Summary of plant and soil chemical and botanical analysis

Sample	Ca^2+a,b^	Mg^2+^	K^+^	Na^+^	N − NH _4_^+^	N − NO _3_^−^	T–N	P − PO _3_^4 −^	*C*_leaf_ (%)	*N*_leaf_ (%)	*δ* ^13^C (‰)	Free sugars (%)	Fructan (%)	Age (years)	Rad. growth (mm)	Mycorrhiza/DSE^e^
Soil	3423^c^	3735	1447	132	6.2	1.4	471	24.1								
*S. gnaphalodes*	2427	3363	1018	141	1.6	0.8	203	25.1	43.3	3.2	−25.2	3.2	12.8	4.7	0.08	0
*W. tridactylites*	2933	5684	1425	162	0.8	1.1	313	27.6	39.5	1.9	−26	17.7	0.8	5.7	0.07	0
*L. klimesii*	2469	3373	1345	168	0.1	0.7	210	22.6	37.3	2	−25.8	4.3	0.2	15.3	0.05	0
*D. altaica*	3108	3765	1545	156	1.5	2.6	438	23	38	3.1	−27.4	5.6	4.4	8	0.04	0
*P. attenuata*	2747	3130	1456	202	1.6	10.7	651	23.6	44	1.1	−26.5	1.3	6.5	–	–	1
*D. alshehbazii*	3528	3548	1862	164	1	2	405	23.9	–^d^	–	–	–	–	–	–	0

^a^Units are milligrams per kilogram dry soil, unless otherwise mentioned

^b^Ca^2+^, Mg^2+^, K^+^, Na^+^, N − NH _4_^+^, N − NO _3_^−^, T–N and P − PO _3_^4 −^ were measured in the soil (rhizospheric and bare), whilst the contents of plant carbon, nitrogen, free sugars, and fructan and the *δ* ^13^C (in per mille) were measured in the plant tissue

^c^Values are means. For the bare soil samples, *n* = 6, whilst for the plants, *n* = 3

^d^Could not be determined

^e^Number of distinct morphotypes

### Anatomical Properties of the Plants

Many common anatomical features were recorded in the studied plants. *L. klimesii*, *D. altaica*, *W. tridactylites* and *S. gnaphalodes* are perennials and have secondary growth and growth or annual rings. The plants had very small rings (0.03–0.05 mm), which is typical of plants growing under limiting environmental conditions. Common to these species are thick-walled, small and short vessels (10–30 μm in diameter and 40–150 μm in length), with simple perforation plates and scalariform to reticulate intervessel pits and absent fibres. Vessel walls are hardly lignified, except in *D. altaica*. A thick bark is also characteristic of all these species. Dating of *L. klimesii*, *D. altaica* and *W. tridactylites* was done by counting the number of rings in their taproots. In *S. gnaphalodes*, age determination was done by stem ring counts. Due to early decay of the xylem and stem reformation in this species, the number of rings represent the age of the oldest remaining tissue. Ring counting dating was not possible in *P. attenuata* since this species does not produce annual rings and also not for *D. alshehbazii* due to the plants’ young age. Number of rings ranged from 4.7 to 15.3 on average, with the exception of one individual of *L. klimesii* representing 22 rings. These counts were used as estimates of the plant ages, although it cannot be excluded that ring counting underestimates plant age because radial growth may not occur in extremely cold summers. Lastly, the grid-line intersection light microscopy method indicated that none of the plants were associated with AMF, although the roots of *P. attenuata* were intensively colonised by a dark septate fungi (DSE).

### Richness and Diversity Patterns of Soil and Root-Associated Bacteria

**Fig. 2:**
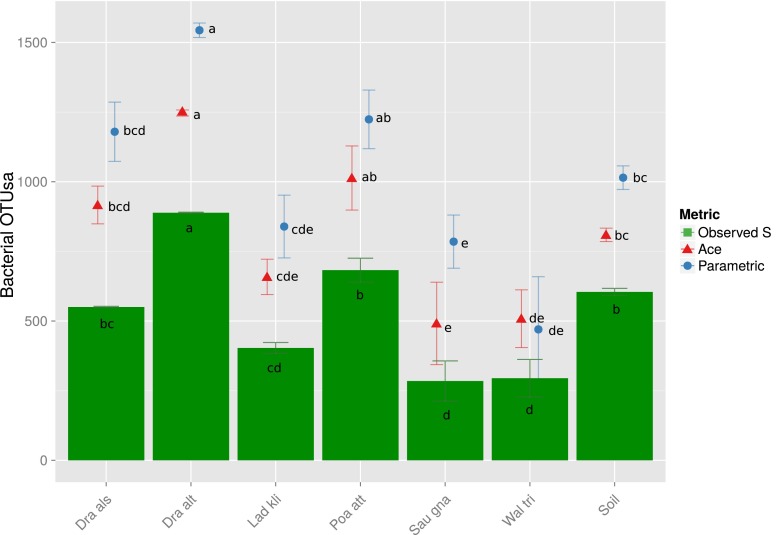
Observed richness (*S*), a non-parametric richness estimation (Ace) and a parametric richness estimation (using CathAll). Means and standard errors in the different plant species and the soil sample. Errors were propagated from the standard errors associated with each replicate. Values were calculated for each sample using bootstrapped subsampling at 1000 iterations. Samples sharing one of the letters appearing next to the symbols are not significantly different (*p* < 0.05)

**Fig. 3:**
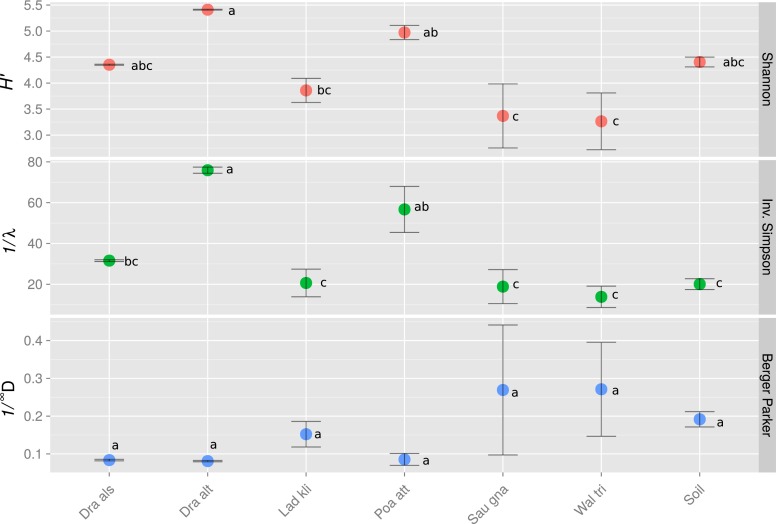
Shannon–Wiener, inverse Simpson and Berger–Parker diversity indices. Means and standard errors in the different plant species and the soil sample. Errors were propagated from the errors associated with each replicate. Values were calculated for each sample using bootstrapped subsampling of the sequences at 1000 iterations. Samples sharing one of the letters appearing next to the symbols are not significantly different (*p* < 0.05)

Out of 721,114 raw sequences, 318,290 passed quality control, of which 7550 were unique (Table 2). Following subsampling (to normalise read depth), the sequences were grouped into 2556 OTUs based on 97 % sequence similarity and average neighbour joining. The number of OTUs per sample ranged between 285 ± 115 and 889 ± 2 (mean ± SEM) in the root-associated samples. Despite our expectations, the bare soil samples, which had on average 605 ± 13 OTUs, were not the richest amongst the samples; rather, these were the root communities of *D. altaica* and *P. attenuata*, with 889 ± 2 and 683 ± 43 observed OTUs, respectively. Since rarefaction analysis showed insufficient sampling depth (Fig. S3), we used parametric and non-parametric richness estimators to estimate sample richness (Fig. 2). For each sample, 150–300 additional OTUs per sample were predicted by the Ace estimator whilst the parametric estimator predicted up to 700 additional OTUs, but both showed similar trends. The bare soil samples were predicted to be poorer than *D. altaica* and about as rich as *P. attenuata* and *D. alshehbazii*. The bare soil samples also did not harbour the most diverse microbiome from all the samples (Fig. 3). Their inverse Simpson index was significantly lower than that of *D. altaica* and *P. attenuata*, whilst their Berger–Parker dominance was one of the highest (although the differences between the samples were insignificant), indicating low diversity and a high dominance of the most abundant OTU. Differences in the Shannon–Wiener index between the bare soil and each of the plant samples were insignificant, but the bare soil nevertheless was the third highest amongst the samples. Reflecting the patterns of observed and predicted richness, the two richest root-associated microbiomes of *D. altaica* and *P. attenuata* were also the most diverse. They were in turn followed by *D. alshehbazii*, *L. klimesii*, *S. gnaphalodes* and *W. tridactylites* in a decreasing order of diversity.

**Table 2:** Summary of sequencing statistics and richness metrics (mean ± SE, *n* = 3)

	*D. alshehbazii*	*D. altaica*	*L. klimesii*	*P. attenuata*	*S. gnaphalodes*	*W. tridactylites*	Soil	Total
Raw	34,030 ± 1054	33,024 ± 2517	22,238 ± 746	18,599 ± 779	21,853 ± 4414	25,129 ± 2632	42,748 ± 2813	721,114
Passed QC	13,192 ± 1055	13,339 ± 1577	9647 ± 1445	11,541 ± 1185	10,214 ± 4570	11,855 ± 3442	18,155 ± 1077	318,290
Unique	913 ± 21	1498 ± 56	626 ± 10	999 ± 38	461 ± 115	496 ± 101	1086 ± 47	7550
OTUs	598 ± 15	985 ± 32	407 ± 17	712 ± 34	384 ± 42	311 ± 67	711 ± 21	2671
Subsampled OTUs	550 ± 2	889 ± 2	403 ± 20	683 ± 43	285 ± 72	294 ± 94	605 ± 13	2556

### Taxonomic Classification of the Bacterial Rhizospheric Communities

**Fig. 4:**
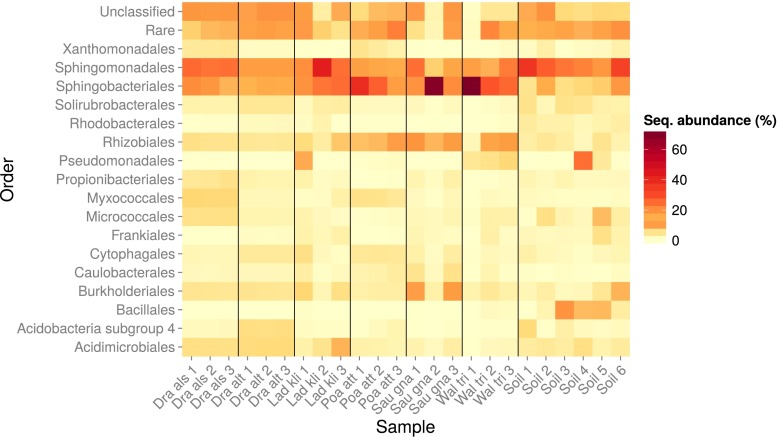
Taxonomic classification and relative abundances of sequences recovered from the root and soil samples. Representative sequences of each OTU classified at the bacterial order level. *Colours* of the heat map indicate the cumulative relative abundance of all sequences in a sample classified to a particular order. Grouping (*bold lines*) separates each sample, whilst *numbers on the X-axis* indicate replicates. Unclassified: sequences with <70 % classification agreement. Rare: all taxa comprising each <1 % of the sequences

The root-associated and soil bacterial communities were relatively simple in terms of phylogenetic diversity on the order level, with the OTUs being classified primarily into five to eight major orders (Fig. 4). About 8 % of the reads in each community could not be confidently assigned to any microbial group at the order level, and about 12 % of the reads from each community were classified as ‘rare’ orders (orders comprising <1 % of the total reads). Sphingomonadales was the only order that was found in high abundance in both the root-associated and soil communities, whilst Acidimicrobiales, Burkholderiales and Sphingobacteriales were also common in the soil and on most roots, but to a lesser extent. The root-associated communities also contained OTUs classified as Rhizobiales in high abundances, but these were relatively rare in the soil samples. Some differences in phylogenetic diversity were also apparent between the root-associated communities. For example, the following orders were abundant in some plants but not others: the order Micrococcales comprised about 5.6 % of the community in *D. alshehbazii*, Myxococcales comprised about 7.1 % of the community in *D. alshehbazii* and about 5.5 % in *P. attenuata*, Acidobacteria subgroup 4 comprised about 6 % of the community in *D. altaica* and was also abundant in some soil samples, *Cytophagales* comprised about 4 % of the community in *D. altaica* and 4 % of the communities in *P. attenuata*, Solirubrobacterales comprised about 4 % of the community in *D. altaica* and was also common in some soil samples, Pseudomonadales comprised as much as 9.4 % of the community in one of the *L. klimesii* samples (but was nearly absent in the others) and about 4.4 % of the communities in *W. tridactylites* (in addition to being very prominent in one of the soil samples), and Xanthomonadales comprised about 4.3 % of the communities in *D. alshehbazii* and about 4 % of the communities in *P. attenuata*.

### Identification of Bacterial Metacommunities

**Fig. 5:**
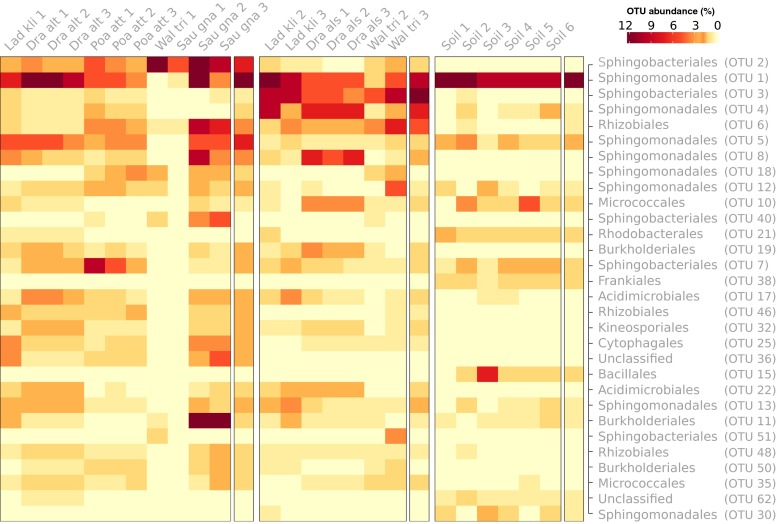
Sample clustering to metacommunities together with a heat map of the root-associated and soil bacterial OTU dataset with hierarchical clustering. Heat map shows the root-associated and soil bacterial OTU abundances (square-root-transformed), with samples grouped according to the metacommunity from which they most likely have originated. The mean of the Dirichlet component for that mixture is shown *to the right of each metacommunity*. Only the first 30 OTUs are shown, those with the greatest variability across metacommunities (see Table S2 for details)

Using Dirichlet multinomial mixtures modelling, the bare soil and root-associated communities were clustered into three metacommunities, from which the samples have supposedly originated (Fig. S4). The three metacommunities had a total mean absolute difference to the reference metacommunity (single component) of 210 %, split 58, 67, and 84 % across components, indicating substantial differences in community structures for each component from the reference. The first metacommunity included all the root-associated communities of *D. altaica*, *P. attenuata* and *S. gnaphalodes*, in addition to one of the *L. klimesii* communities and one of the *W. tridactylites* communities (Fig. 5). This metacommunity had the largest contribution to the mean metacommunity ($\overline{\pi}=0.458$) and was highly variable ($\overline{\theta}=506$). The second metacommunity included the remaining two root-associated communities of *L. klimesii* and *W. tridactylites*, in addition to all the rhizosphere communities of *D. alshehbazii*, and had a weight of 0.292 and a variability of 364. The third metacommunity included only the soil samples. It contributed least to the mean metacommunity ($\overline{\pi}=0.25$), but was the most variable ($\overline{\theta}=635$). All three metacommunities are predicted to be dominated by a single OTU assigned as a Sphingomonadales member, with estimated means of 4.65 and 6.63 % in the two rhizophere metacommunities and with 17 % in the soil metacommunity (Table S2), but differentiate in the identity or importance of other dominating OTUs. Primarily, the soil communities, nearly or entirely, lacked two major OTUs assigned as Sphingobacteriales, which were of high abundance in all root-associated communities and accounted for an estimated 12.1 and 0.73 % and 0.4 and 9 % for the first and third OTUs in metacommunities 1 and 2, respectively. Additional notable differences were the presence of dominant Rhizobiales and Burkholderiales OTUs, as well as several OTUs assigned to Sphingomonadales, on the roots but not in the soil. In contrast, Frankiales and Bacillales were found almost exclusively in the bare soil communities.

## Discussion

Here, we report on the occurrence of six vascular plants at an unprecedented altitude of 6150 m.a.s.l. and characterise their habitat and their root-associated and bare soil bacterial community. The six plant species were all growing under highly stressful conditions, but nevertheless indicated stable growth. Out of the four plant species for which age could be determined, three were very young (<10 years), whilst one (*L. klimesii*) was 15 years on average, providing evidence to the recent upward migration of the plants and the rapid changes these Western Himalayan slopes undergo. Nevertheless, the monitored temperature and snow cover profiles indicated that the plants could probably utilise no more than a few weeks per year for growth. The high *δ*^13^C values, which are typical of plants growing at high altitude [16], indicate high water use efficiency imposed by stress conditions caused by drought and frost. In addition, the high concentrations of fructan in *L. klimesii* and *W. tridactylites* and soluble sugars in *W. tridactylites* and *D. altaica* could also indicate growth retardation (but could also serve as compatible solutes, combating water deficit caused by drought or frost) [49, 50]. Only minor changes in the chemical composition of the rhizosphere soil could be observed compared to the bare soil. Most notably, a decrease in ammonia concentrations was observed, which is either due to the presence of biological soil crusts or the high uptake by plants.

Despite their young age and the poorly developed soil, the plants harboured several hundred observable OTUs on their roots (with more than 1000 predicted), with community richness not correlating to plant age (data not shown). Surprisingly, other studies performing amplicon sequencing of rhizosphere and rhizoplane bacteria on divergent plants ranging from wild plants growing on the shores of an Antarctic bay [51] to cultivated rice [52] and maize [53] growing on fully developed soils have reported similar richness values (approx. 300–1000 OTUs), demonstrating the remarkable ability of microbes to saturate the niches around the roots even in such harsh and poor environment.

As expected from a young and poorly developed soil, it was poorer in OTUs compared to most other studied mature soils, which typically harbour 800–2000 observed OTUs and up to 5000 estimated total OTUs (e.g. [54, 55]), although it was similar to or even richer than other mature desert soils [56–58]. To our surprise, the soil communities showed similar richness to the rhizoplane communities and were even poorer than the communities on the roots of *P. attenuata* and *D. altaica*. This contrasts our initial hypothesis that the soil acts as a microbial reservoir from which the plant roots recruit their associates.

All root-associated communities were dominated by a similar and relatively small number of bacterial orders. Superficially, the community composition of the root-associated samples was similar between plants and resembled that of the bare soil. All communities were dominated primarily by members of the order Sphingomonadales (Alphaproteobacteria), whilst the root samples also had high proportions of Sphingobacteriales (Bacteroidetes), Rhizobiales (Alphaproteobacteria) and Burkholderiales (Betaproteobacteria). On the class level (i.e. alpha- and betaproteobacteria, and Bacteroidetes), these bacteria are typical of root environments; however, many of the orders are uncommon. Together with the high proportions of Actinobacteria (e.g. Solirubrobacterales and Propionibacteriales) and the low proportions of Acidobacteriales (Acidobacteria), the bare soil and root-associated communities were highly typical of soils and crusts from hot or arctic deserts [59–61], but very different from the soil or root-associated communities found at high-altitude temperate regions [62–64]. The latter were more diverse on the class level and were characterised by high proportions of Acidobacteria, gamma- and deltaproteobacteria and Planctomycetes and consecutively lower proportions of alpha- and betaproteobacteria and Actinobacteria. In particular, Sphingomonadales and Sphingobacteriales were also found to dominate and be very active in biological soil crusts from the Negev Desert [56] and in soils recovered from the Atacama [65] and Death Valley deserts [66], but to our knowledge have never been reported as the dominant members of root-associated communities. This demonstrates that whilst subnival zones and glacial forefields select for specifically adapted bacteria [67], the resulting bacterial community nevertheless resembles that of the surrounding ecosystem (i.e. arid), which acts as the major source of inoculation [68].

Root-associated bacterial communities play diverse roles in helping plants establish. Since all were devoid of associated mycorrhiza, the plants must have relied on prokaryotes for support in acquiring minerals. Biologically fixed nitrogen is one of the most limiting factors in newly deglaciated soils and, consequently, also one of the first microbial processes to establish in such sites [69]. Pioneer plants have also been shown to harbour active diazotrophs on their roots, displaying an overall higher population density and activity than the surrounding soil [64, 70]. As mentioned above, the presence of members of Rhizobiales on the plant roots but not in the soil is a strong indication of root-associated diazotrophy. In addition, whilst to our knowledge never directly demonstrated, members of the order Sphingomonadales possess the nitrogenase gene and could possibly act as root-associated diazotrophs (in contrast to symbiotic diazotrophs) [71]. Sphingomonadales are also known to utilise a wide range of naturally occurring compounds including root exudates and are commonly found on plant roots [72, 73]. Sphingobacteriales have also been found on the roots of some plants in the past and have been shown to utilise root exudates [72]. Members of this order are heterotrophs known to utilise a wide variety of carbon sources [74] and are also often detected in hydrocarbon-contaminated soils [75].

The extent to which the root-associated bacterial community is contingent with specific plant species is still debated. Various studies, particularly on cultivated plants, have found large variations in community structure based on the plant species studied (e.g. [72, 76, 77]). Yet in many field studies where wild plants growing in vicinity were studied, no significant differences in bacterial communities between plant species were detected, or they were very small. For example, Nunan and colleagues [78] studying grasslands in the UK and Teixeira and colleagues [51] studying vascular plants in the Antarctic were unable to cluster root-associated bacteria by plant species, indicating that, for wild plants growing under natural conditions, the environment plays a much stronger role in determining the associated community than the plant species. In this study, the main bacterial orders were shared between nearly all plants, yet the root-associated communities were clustered into two distinct metacommunities. An examination of these metacommunities shows that they mostly split based on the relative abundance of some dominant Sphingobacteriales and Sphingomonadales OTUs and not on presence or absence. Since the possible physiological differences between these OTUs are unknown, it remains unclear whether these differences can be attributed to more than stochastic colonisation processes.

Clear differences were however found between the root-associated communities and the surrounding soil, separating them into distinct metacommunities. Whilst both the plant and bare soil metacommunities were dominated by members of the same bacterial orders, many of the dominant OTUs in the plants (most notably OTUs 2, 3, 8, 18 and 40) were missing in the soil. Some of these bacteria, such as Rhizobiales, are well known for forming root associations and are expected to be absent or nearly absent in soil, whilst others such as Sphingobacteriales and Sphingomonadales were dominant in both soil and root communities, but were represented by different OTUs, which could differ significantly in their ecophysiology. Likewise, some highly ranked OTUs in the soil communities were absent in the root-associated ones (most notably OTU 15 classified as Bacillales and OTU 38 classified as Frankiales). This stands in contrast to previous reports on the rhizospheric microbial community of pioneer plants in glacial forefields (e.g. [79–81]) which found no difference between soil and root-associated communities. However, this discrepancy could probably be explained by the use of a more sensitive and robust community profiling technique in this work (high-throughput sequencing compared to molecular fingerprinting techniques). Whilst the complete absence of these dominant root-associated OTUs in the soil cannot be fully proven, the differences between root and bare soil communities, together with the fact that the soil was not richer than several of the plants, imply that some bacteria were transported on, or in, the dispersing plant seeds and later became part of the root-associated microbial community. The fact that plant seeds harbour a diverse and viable microbial community on and in them is evident from numerous studies [82]. Moreover, studies have shown that seed-borne bacterial communities tend to resemble those of the soil from which the mother plant was grown [83] and that some of these bacteria (including also certain rhizobia) make their way later on to the root surface and rhizosphere of the germinating plants [83–85]. The transport and later proliferation of certain bacteria on the roots, following germination, could explain the source of the OTUs not present in the surrounding soil. This indicates that pioneer plants do not only recruit microbes from the surrounding soil but also enrich the soil with allochthonous bacteria and help establish a mature bacterial soil community.

## Electronic supplementary material

Below is the link to the electronic supplementary material.

Fig. S1 (DOCX 1991 kb)

Fig. S2 (DOCX 438 kb)

Fig. S3 (DOCX 155 kb)

Fig. S4 (DOCX 129 kb)

Table S1 (DOCX 18 kb)

Table S2 (DOCX 22 kb)
