# Supplementary material for: The Root-Associated Microbial Community of the World’s Highest Growing Vascular Plants
Source: Microb Ecol. 2016 May 31;72:394–406. doi: 10.1007/s00248-016-0779-8 (PMC4937074; doi:10.1007/s00248-016-0779-8)
Supplement: Supplementary file 1 — (DOCX 1991 kb) [file 248_2016_779_MOESM1_ESM.docx]

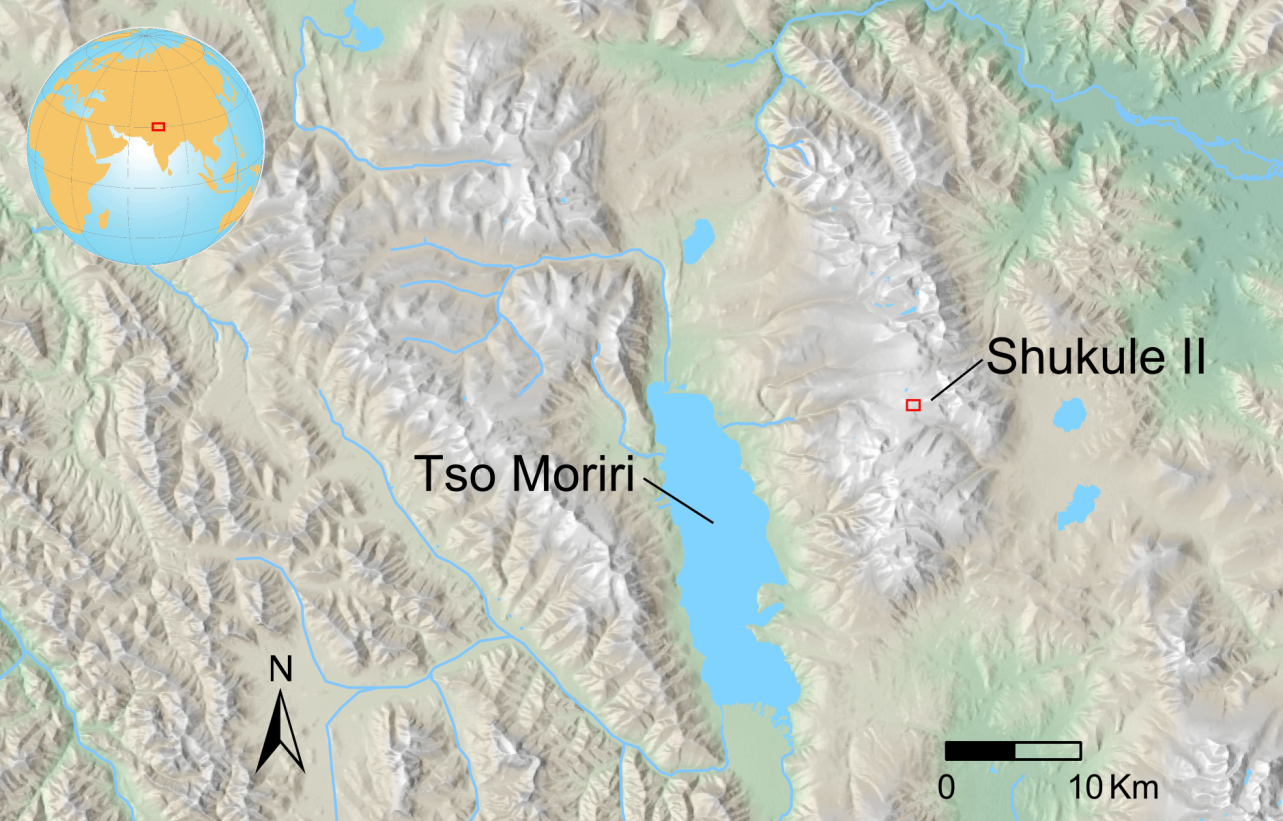


**Supplementary Figure 1. A map of the studied region in the Western Himalayas showing the sampling site near the top of mount Shukule II.**
