# Supplementary material for: The Root-Associated Microbial Community of the World’s Highest Growing Vascular Plants
Source: Microb Ecol. 2016 May 31;72:394–406. doi: 10.1007/s00248-016-0779-8 (PMC4937074; doi:10.1007/s00248-016-0779-8)
Supplement: Supplementary file 2 — (DOCX 438 kb) [file 248_2016_779_MOESM2_ESM.docx]

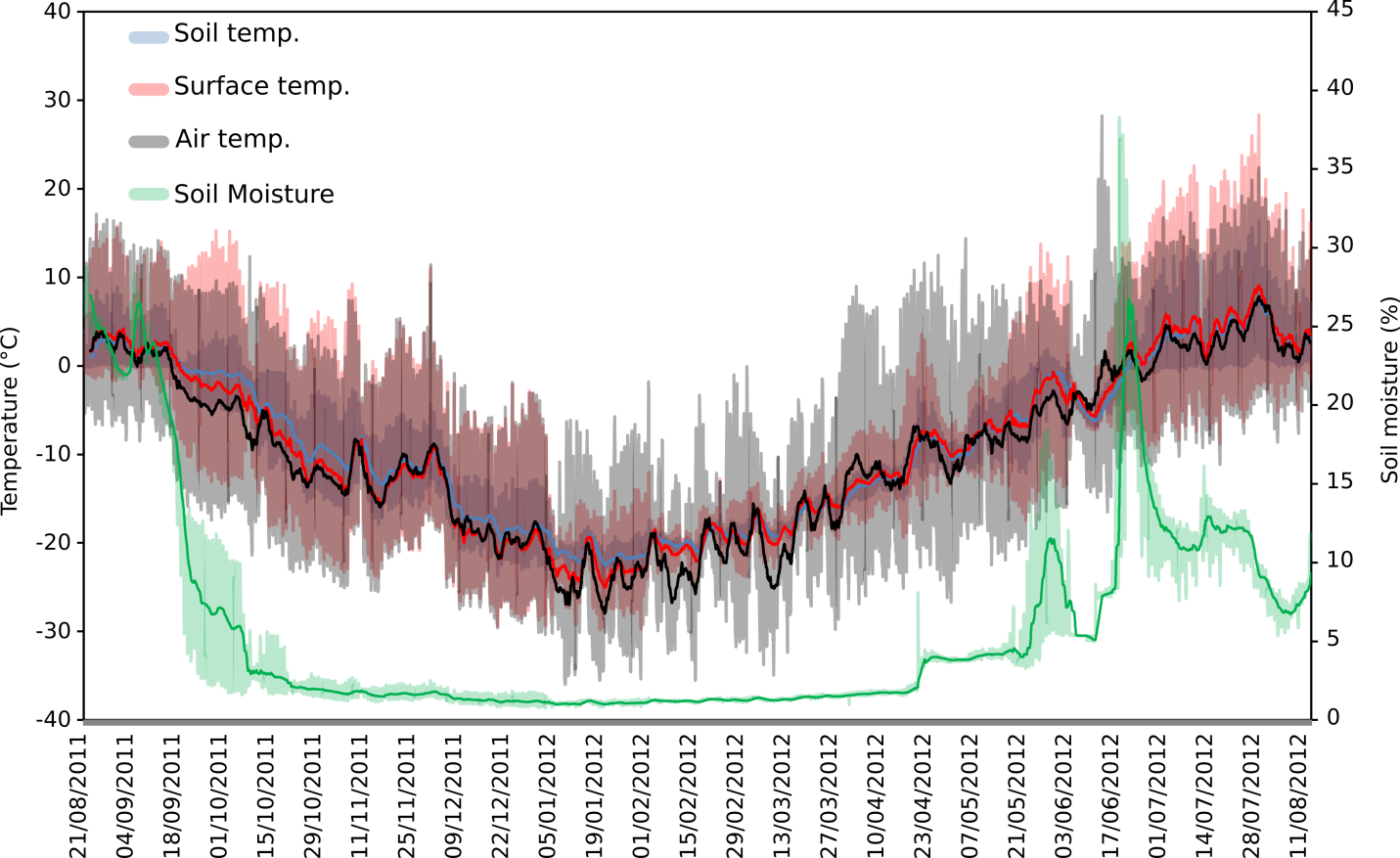


**Supplementary Figure 2.** **Daily course of air, surface and soil temperatures, and soil moisture measured at an elevation of 6150 masl on the south-western slope of Shukule II Peak in Tso Moriri Area, East Ladakh between August 2011 and August 2012.**
