# Supplementary material for: The Root-Associated Microbial Community of the World’s Highest Growing Vascular Plants
Source: Microb Ecol. 2016 May 31;72:394–406. doi: 10.1007/s00248-016-0779-8 (PMC4937074; doi:10.1007/s00248-016-0779-8)
Supplement: Supplementary file 3 — (DOCX 155 kb) [file 248_2016_779_MOESM3_ESM.docx]

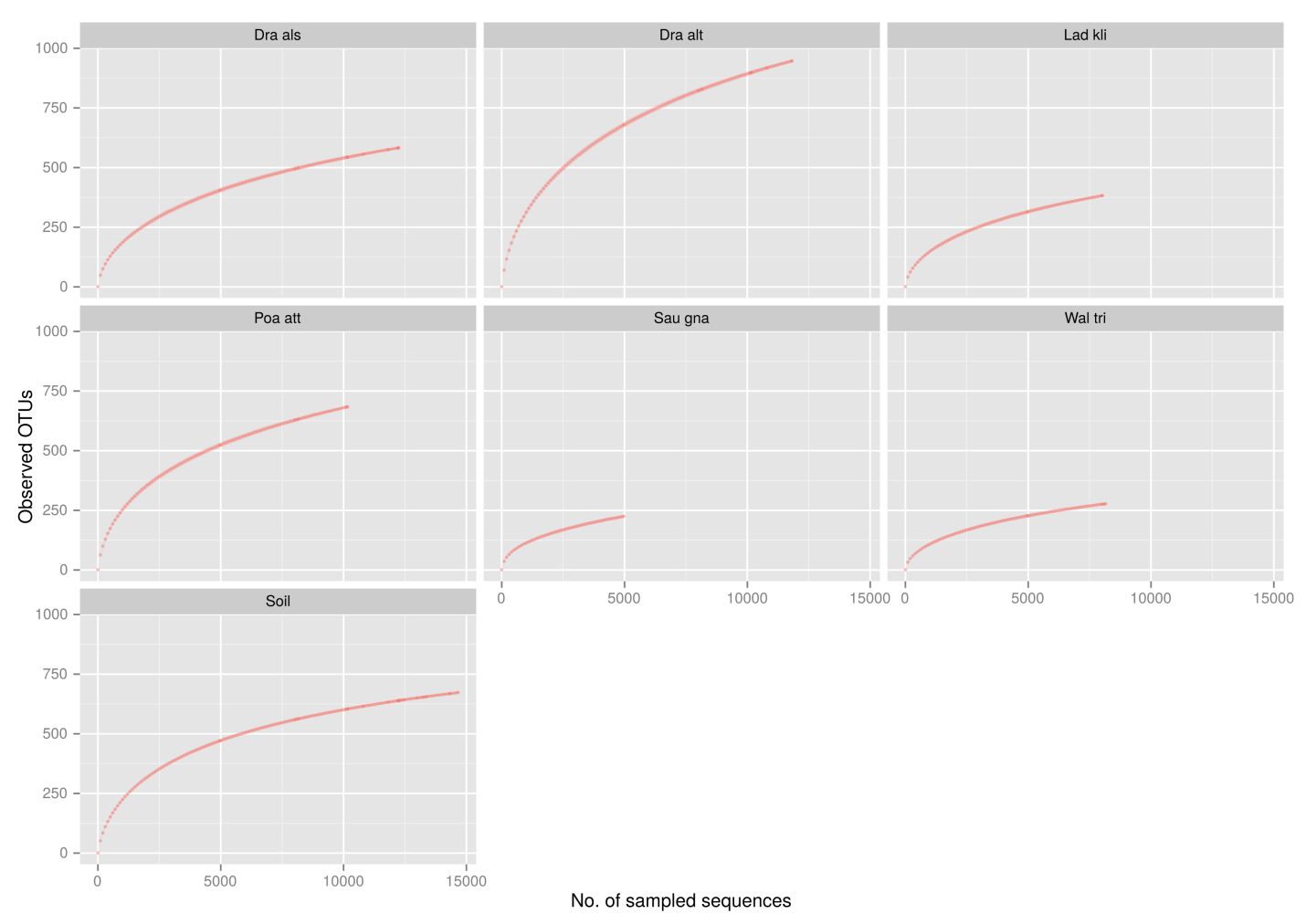


**Supplementary Figure 3.** **Rarefaction curves: accumulation of observed OTUs with increasing sampling effort (number of sequences).** OTUs were defined based on 97% sequence identity and the average neighbour clustering algorithm. Error bands are propagated 95% confidence intervals. The curves were generated each using a 1000 iterations.
