# Supplementary material for: The Root-Associated Microbial Community of the World’s Highest Growing Vascular Plants
Source: Microb Ecol. 2016 May 31;72:394–406. doi: 10.1007/s00248-016-0779-8 (PMC4937074; doi:10.1007/s00248-016-0779-8)
Supplement: Supplementary file 4 — (DOCX 129 kb) [file 248_2016_779_MOESM4_ESM.docx]

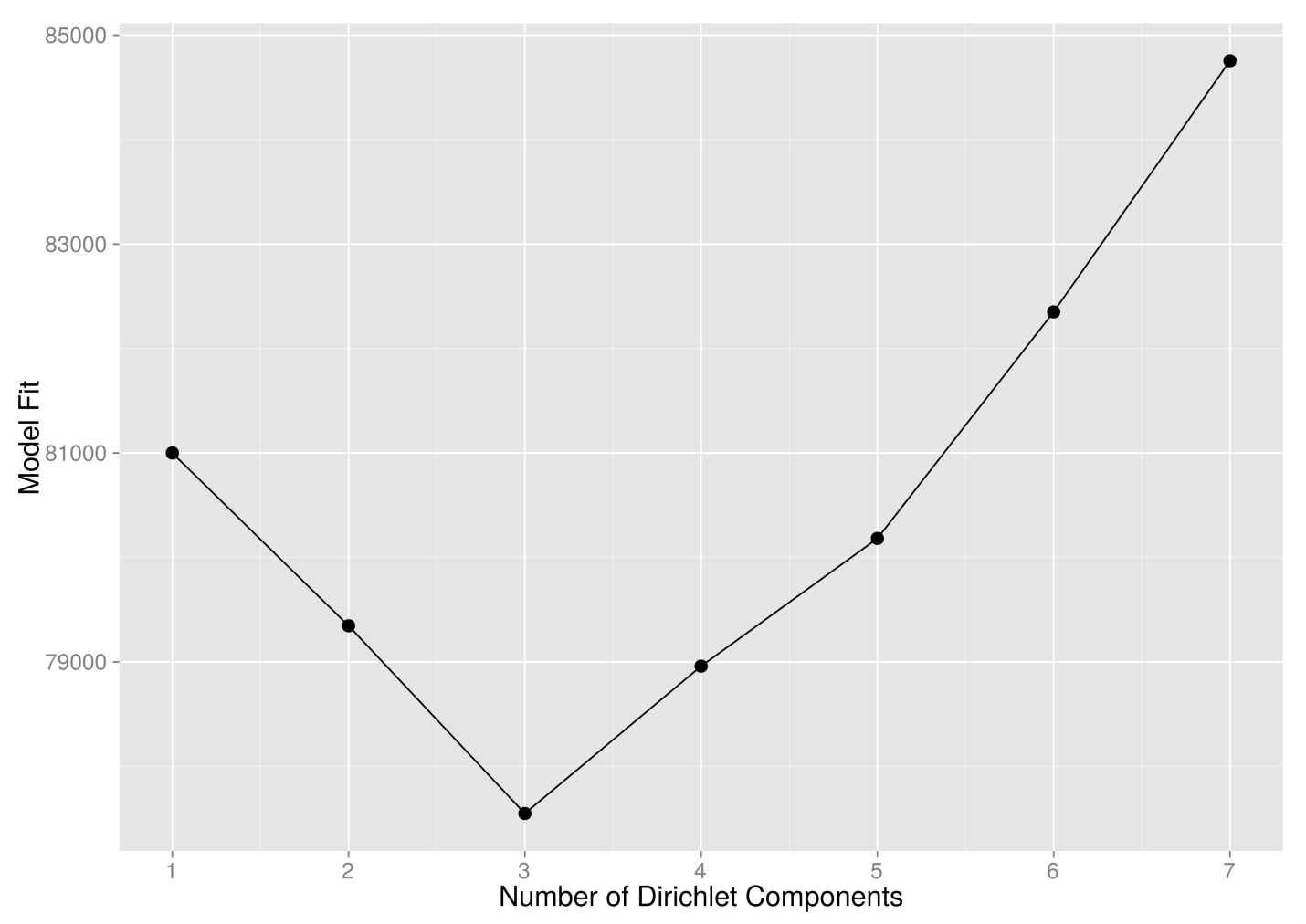


**Supplementary Figure 4.** **Model fit for a mixture of Dirichlets priors to the root-associated and soil bacterial OTU dataset.** Model fit was calculated using the Laplace approximation to the negative log model evidence and is plotted against the number of Dirichlet mixture components tested (K). The minimum indicates the appropriate number of Dirichlet priors to be used in the model, which reflects the number of metacommunities.
