# Supplementary material for: The Root-Associated Microbial Community of the World’s Highest Growing Vascular Plants
Source: Microb Ecol. 2016 May 31;72:394–406. doi: 10.1007/s00248-016-0779-8 (PMC4937074; doi:10.1007/s00248-016-0779-8)
Supplement: Supplementary file 5 — (DOCX 18 kb) [file 248_2016_779_MOESM5_ESM.docx]

**Supplementary Table 1.** Sequencing barcodes used in this study.

| # | Sample | Barcode |
| --- | --- | --- |
| 1 | D. alshehbazii 1 | AAGCTTGC |
| 2 | D. alshehbazii 2 | CTGAGACT |
| 3 | D. alshehbazii 3 | TCAGAGTC |
| 4 | D. altaica 1 | GTGTTGTG |
| 5 | D. altaica 2 | AGAGGAGA |
| 6 | D. altaica 3 | GAAGGAAG |
| 7 | L. klimesii 1 | TAGGCCAT |
| 8 | L. klimesii 2 | ACCACAAC |
| 9 | L. klimesii 3 | TGACACTG |
| 10 | P. attenuata 1 | ACCAACCA |
| 11 | P. attenuata 2 | ACCATGGT |
| 12 | P. attenuata 3 | CAACGTTG |
| 13 | S. gnaphalodes 1 | TGCACATG |
| 14 | S. gnaphalodes 2 | TTGCAAGC |
| 15 | S. gnaphalodes 3 | AATACCGC |
| 16 | W. tridactylites 1 | ATATCGCG |
| 17 | W. tridactylites 2 | ATGCATCG |
| 18 | W. tridactylites 3 | GCTTAACG |
| 19 | Soil 1 | TAGGATGG |
| 20 | Soil 2 | ACGTTGCA |
| 21 | Soil 3 | AAGCCGAA |
| 22 | Soil 4 | AGCTCTAG |
| 23 | Soil 5 | CTCTGAGA |
| 24 | Soil 6 | CCTAATGG |
