# Supplementary material for: The Root-Associated Microbial Community of the World’s Highest Growing Vascular Plants
Source: Microb Ecol. 2016 May 31;72:394–406. doi: 10.1007/s00248-016-0779-8 (PMC4937074; doi:10.1007/s00248-016-0779-8)
Supplement: Supplementary file 6 — (DOCX 22 kb) [file 248_2016_779_MOESM6_ESM.docx]

**Supplementary Table 2.** Estimated OTU relative abundances and differences between meta-communities.

|  | ***m_0j*** | ***m_1j*** | ***m_2j*** | ***m_3j*** | **Diff.** | **C. Diff.** |
| --- | --- | --- | --- | --- | --- | --- |
| Sphingobacteriales (OTU 2) | 0.7 | 12.1 | 0.7 | 0 | 55.7 | 16.3 |
| Sphingomonadales (OTU 1) | 6.9 | 4.7 | 6.6 | 17 | 17.6 | 21.5 |
| Sphingobacteriales (OTU 3) | 0.6 | 0.4 | 9.4 | 0 | 9.7 | 24.3 |
| Sphingomonadales (OTU 4) | 0.9 | 0.4 | 4.7 | 0.7 | 5.3 | 25.9 |
| Rhizobiales (OTU 6) | 1.3 | 1.6 | 3.6 | 0.2 | 4.3 | 27.2 |
| Sphingomonadales (OTU 5) | 2 | 2.7 | 1 | 2.4 | 3.8 | 28.3 |
| Sphingomonadales (OTU 8) | 0.5 | 1.3 | 2 | 0 | 3.3 | 29.3 |
| Sphingomonadales (OTU 18) | 0.3 | 0.9 | 0.1 | 0.1 | 3 | 30.1 |
| Sphingomonadales (OTU 12) | 0.9 | 1.4 | 0.5 | 0.8 | 2.8 | 31 |
| Micrococcales (OTU 10) | 0.5 | 0.2 | 1.1 | 1.8 | 2.6 | 31.7 |
| Sphingobacteriales (OTU 40) | 0.1 | 0.5 | 0.1 | 0 | 2.2 | 32.4 |
| Rhodobacterales (OTU 21) | 0.3 | 0.1 | 0.2 | 1.9 | 2.1 | 33 |
| Burkholderiales (OTU 19) | 0.5 | 0.7 | 1.3 | 0.1 | 1.8 | 33.5 |
| Sphingobacteriales (OTU 7) | 1.2 | 0.9 | 1.2 | 1.9 | 1.8 | 34 |
| Frankiales (OTU 38) | 0.2 | 0.1 | 0.1 | 1.5 | 1.6 | 34.5 |
| Acidimicrobiales (OTU 17) | 0.7 | 1 | 0.9 | 0.2 | 1.5 | 34.9 |
| Rhizobiales (OTU 46) | 0.4 | 1 | 0.3 | 0.1 | 1.5 | 35.4 |
| Kineosporiales (OTU 32) | 0.4 | 0.7 | 0.8 | 0.1 | 1.4 | 35.8 |
| Cytophagales (OTU 25) | 0.3 | 1.1 | 0.4 | 0 | 1.3 | 36.2 |
| Unclassified (OTU 36) | 0.1 | 1 | 0 | 0 | 1.3 | 36.6 |
| Bacillales (OTU 15) | 0.1 | 0 | 0 | 1.3 | 1.3 | 37 |
| Acidimicrobiales (OTU 22) | 0.4 | 0.4 | 1 | 0.1 | 1.2 | 37.3 |
| Sphingomonadales (OTU 13) | 1 | 0.9 | 1.3 | 0.6 | 1.1 | 37.7 |
| Burkholderiales (OTU 11) | 0.7 | 0.7 | 0.6 | 0.6 | 1.1 | 38 |
| Sphingobacteriales (OTU 51) | 0 | 0.2 | 0 | 0 | 1.1 | 38.3 |
| Rhizobiales (OTU 48) | 0.4 | 0.6 | 0.6 | 0.1 | 1.1 | 38.7 |
| Burkholderiales (OTU 50) | 0.2 | 0.6 | 0.2 | 0 | 1.1 | 39 |
| Micrococcales (OTU 35) | 0.5 | 0.7 | 0.8 | 0.1 | 1 | 39.3 |
| unclassified (OTU 62) | 0.2 | 0.1 | 0.2 | 0.8 | 1 | 39.6 |
| Sphingomonadales (OTU 30) | 0.1 | 0 | 0 | 0.9 | 0.9 | 39.8 |

Estimated relative abundances of 30 OTUs which contributed mostly to the the total mean difference between the the three Dirichlet mixture components ($\overline{m}_{1}...\overline{m}_{3}$) fitted to the data and the estimate of the mean of the reference single Dirichlet component ($\overline{m}_{0}$). OTUs are ranked in order of their contribution to the total mean difference of 210%, split 58%, 67%, and 84% across components. Diff: mean difference of each OTU. C.Diff: cumulative fraction of this difference accounted for.
